# Antibacterial Activities and Molecular Docking of Novel Sulfone Biscompound Containing Bioactive 1,2,3-Triazole Moiety

**DOI:** 10.3390/molecules26164817

**Published:** 2021-08-09

**Authors:** Huda R. M. Rashdan, Ihsan A. Shehadi, Mohamad T. Abdelrahman, Bahaa A. Hemdan

**Affiliations:** 1 Chemistry of Natural and Microbial Products Department, Pharmaceutical and Drug Industries Research Division, National Research Centre, Dokki, Cairo 12622, Egypt; 2 Chemistry Department, College of Science, University of Sharjah, Sharjah 27272, United Arab Emirates; ishehadi@sharjah.ac.ae; 3 Radioisotopes Department, Nuclear Research Centre, Egyptian Atomic Energy Authority, Cairo 12311, Egypt; mohamadt.abdelrahman@gmail.com; 4 Water Pollution Research Department, Environmental Research Division, National Research Centre, 33 El Buhouth Street, Cairo 12622, Egypt; be.hemdan@iitg.ac.in

**Keywords:** 1,2,3-triazoles, dapsone, sulfone biscompounds, antibacterial activity, MIC, ATP level, toxicity

## Abstract

In this study, a new synthetic 1,2,3-triazole-containing disulfone compound was derived from dapsone. Its chemical structure was confirmed using microchemical and analytical data, and it was tested for its in vitro antibacterial potential. Six different pathogenic bacteria were selected. MICs values and ATP levels were determined. Further, toxicity performance was measured using MicroTox Analyzer. In addition, a molecular docking study was performed against two vital enzymes: DNA gyrase and Dihydropteroate synthase. The results of antibacterial abilities showed that the studied synthetic compound had a strong bactericidal effect against all tested bacterial strains, as Gram-negative species were more susceptible to the compound than Gram-positive species. Toxicity results showed that the compound is biocompatible and safe without toxic impact. The molecular docking of the compound showed interactions within the pocket of two enzymes, which are able to stabilize the compound and reveal its antimicrobial activity. Hence, from these results, this study recommends that the established compound could be an outstanding candidate for fighting a broad spectrum of pathogenic bacterial strains, and it might therefore be used for biomedical and pharmaceutical applications.

## 1. Introduction

As the resistance of pathogenic bacteria to the available antibiotics is rapidly becoming a significant international challenge, the development of new compounds to combat antibiotic resistance is among the most significant facets of preliminary antimicrobial research. In particular, it is recognized that antibacterial medications do not even have specific potency, regardless of the biological similarity between human cells and types of pathogens [1].

A broad range of pathways are involved in the emergence of resistant bacteria, including the genetic modifications of horizontal genome transmission and alterations [2]. Additionally, the excessive consumption and abuse of antibiotics can produce multi-drug resistant bacteria that do not require a prescription, leading to the appearance and deployment of antibiotic resistance [3]. Fundamental features have been ascertained in various bacterial strains, encouraging them to resist and block antibiotic attacks [4]. The isolated strains of *Staphylococcus aureus* possess significant resistance to many types of antibiotics, including lactams, glycopeptides, aminoglycosides, and fluoroquinolones [5]. Moreover, *Pseudomonas aeruginosa* is distinguished by its capability to persist in highly antibiotic-resistant biofilm accumulation [6].

Consequently, a large number of studies should be devoted to producing new antibacterial drugs with entirely different chemical formulations and specific potential applications [7]. For some compounds with 1,2,3-triazole, their antimicrobial activity has been recognized [8,9,10,11,12,13]. Dapsone, a sulphone analog known as diaminodiphenyl sulfone (DDS), is a standard antibiotic widely used in combination with clofazimine and rifampicin for the treatment of leprosy. It is the second-line medication for the prevention and treatment of pneumocystis pneumonia. Additionally, it can be used to prevent toxoplasmosis in patients with poor immune function. It has also been used for dermatitis herpetiformis, acne, and other skin disorders [14]. It has been documented as an effective antimicrobial agent [15].

## 2. Results and Discussion

### 2.1. Chemistry

**Scheme 1:**
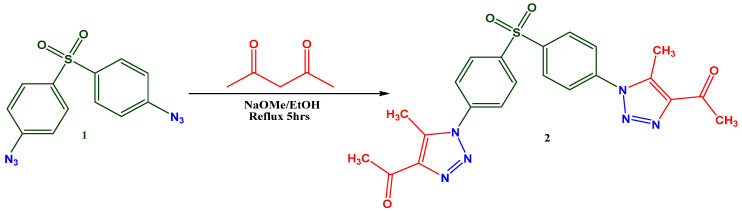
Synthesis of 1,1′-((sulfonylbis(4,1-phenylene))bis(5-methyl-1*H*-1,2,3-triazole-1,4-diyl))bis(ethan-1-one) yield 95% (**2**).

4,4′-sulfonylbis(azidobenzene)(**1**) was submitted to react with acetylacetone in ethanol in the presence of sodium methoxide under reflux to afford the corresponding target compound (Scheme 1). ^1^H NMR spectrum of the product showed characteristic two singlet signals at *δ* 2.36 and 2.42 for the protons of 4-methyl groups, and two douplet signals at 7.75 and 8.11 for the aromatic protons; ^13^C-NMR showed significant signals at *δ* 9.74, 27.69, 126.65, 129.33, 138.10, 139.18, 141.41, 142.98, 193.33; the structure was also supported by its mass spectrum (*m*/*z* (464)) [M+], which agrees with its molecular formula C_22_H_20_N_6_O_4_S (details are presented in the Supplementary Chart 1 and Chart 2).

### 2.2. Biology

In this study, an inhibitory zone assay was applied to assess the antibacterial properties of the newly synthesized compound towards *E.* *coli* O157, *P.* *aeruginosa*, *K.* *pneumonia*, *S.* *aureus*, *B.* *subtilis*, and *L.* *monocytogenes*, using an agar well diffusion assay. The obtained results indicated that the newly synthesized compound displayed significantly higher potency for inhibiting a broad spectrum of examined Gram-negative species, including *E. coli* O157, *P.* *aeruginosa,* and *K. pneumoniae* at a concentration of 20  mg/ mL, with the ZOI being 22 ± 0.14, 23 ± 0.28, and 25 ± 0.23 mm, respectively, using a disc diffusion assay, and the ZOI being 24 ± 0.14, 26 ± 0.24 and 27 ± 0.12 mm, respectively, using a well-diffusion assay (Table 1). Simultaneously, the ZOI values of the compound against the examined Gram-positive species, including *S. aureus, B. subtilis,* and *L. monocytogenes*, were 19 ± 0.26, 17 ± 0.16, and 18 ± 0.22, respectively, using the well-diffusion assay. The obtained results show that Gram-negative bacterial species were more susceptible to the compound than Gram-positive.

The obtained results are in agreement with El Malah et al. [16], who revealed that the novel synthesized 1,2,3-triazoles have potent antibacterial activity against a spectrum of bacterial species, including *P. aeruginosa* and *S. aureus*, with the inhibitory zone diameter being around 25 and 19 mm. On another side, the width of the ZOI around discs was smaller than that around wells. These results are compatible with El Nahrawy et al. (2021a), who exhibited that the width of the ZOI in the well-diffusion assay is larger than the disc diffusion assay. The ZOI width of Vancomycin as a reference drug was less than the ZOI of the newly synthesized compounds **2**. This means that the inhibitory efficacy of the tested compound is higher than the reference drugs (Vancomycin and Ciprofloxacin) [17] used.

### 2.3. Estimation of MIC and IC_50_

**Figure 1:**
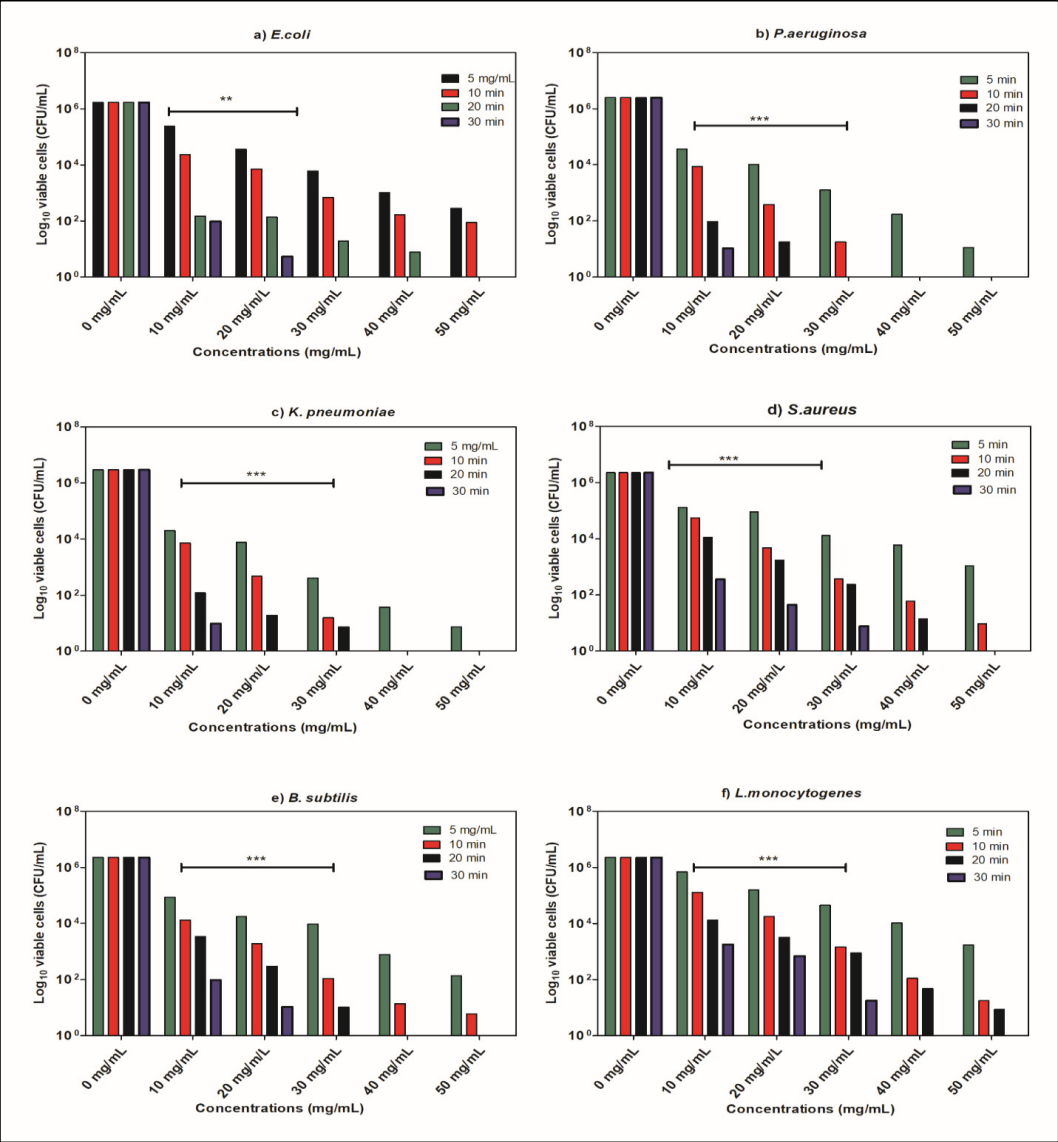
Minimum inhibitory concentration values of the newly synthesized compound towards examined bacterial species (**a**) *E. coli*, (**b**) *P. aeruginosa*, (**c**) *K. pneumonia*, (**d**) *S. aureus*, (**e**) *B. subtilis* and (**f**) *L. monocytogenes*. The remaining viable cell populations at various time intervals of 5, 10, 15, and 30 min are also shown. Two-way analysis of variance (*ANOVA*) states ** indicates moderate correlation (*p* ≤ 0.01), *** indicates high correlation (*p* ≤ 0.001).

Different concentrations of the newly tested compound were then subjected to the MIC test to confirm their antibacterial activities further. Interestingly, as shown in Figure 1, the tested compound revealed good antibacterial effects against all bacteria tested, with different values of MICs depending upon the concentrations (mg/mL) and contact times (min). The results of the MIC test exhibited hat the compound has powerful antibacterial effects against *E.* *coli* (MIC = 40 mg/mL within 20 min), *P.* *aeruginosa* (MIC = 40 mg/mL within 10 min), *K.* *pneumonia* (MIC = 40 mg/mL within 10 min), *S. aureus* (MIC = 50 mg/mL within 20 min), *B. subtilis* (MIC = 40 mg/mL within 20 min) and *L. monocytogenes* (MIC = 40 mg/mL within 30 min). Results noticed that the MIC values of the compound were lower in Gram-negative than in Gram-positive bacteria. The antibacterial activity difference of the tested compound could be found due to variations in chemical composition in bacterial cell walls and its ability to enter the membranes of bacterial cells [18].

Regarding the estimated MIC values for the reference antibiotics drugs (Vancomycin and Ciprofloxacin), the results indicated that *S. aureus* was more susceptible to Vancomycin, while *E. coli* O157 and *P. aeruginosa* were more resistant than the others. On the other hand, *S. aureus* was more resistant to Ciprofloxacin (Table 2).

**Figure 2:**
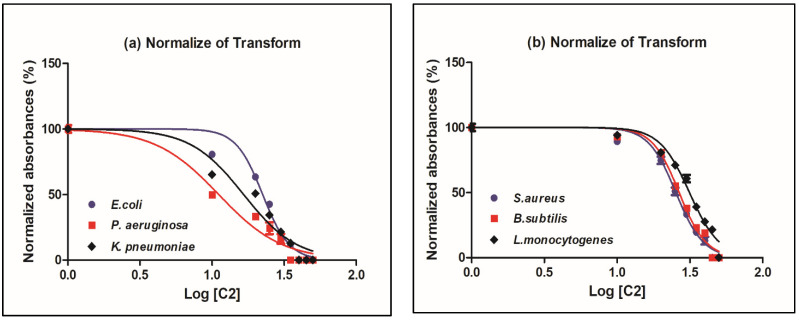
Normalized absorbance (%) of the newly synthesized compound against (**a**) Gram-negative and (**b**) Gram-positive bacteria.

The IC_50_ values for the newly synthesized compound against the investigated bacterial strains are presented in Table 3 and Figure 2. From the IC_50_ value results, the compound appears to act as a vigorous bactericidal agent against the tested bacteria. The IC_50_ values of *E. coli* O157, *P.* *aeruginosa*, *K.* *pneumoniae*, *S.* *aureus*, *B.* *subtilis*, and *L.* *monocytogenes* were 22.42, 11.11, 16.54, 25.31, 26.66, and 31.38 µM, respectively. These results accord with the results of El Nahrawy et al. [19]. In other words, to develop several pharmaceutical coatings expressing anti-HIV, antitumor, and antimicrobial actions, the 1,2,3-triazole dependent heterocycles are well manipulated [13,20,21,22,23].

### 2.4. Kinetic Modeling Using the Pseudo-First-Order Kinetic Model

The pseudo-first-order kinetic model was applied to estimate the inactivation frequencies of the examined bacterial species after their being exposed to the studied compound. The results revealed that the compound could quickly suppress the growth of *P. aeruginosa* from kinetic modeling using the pseudo-first-order model, whereas the minimum inhibition frequency was registered for *L.* *monocytogenes* organisms. Further, the effective concentration of the compound was the one that could efficiently suppress the growth of all bacterial strains studied, depending on the type of bacterial species tested, throughout various retention periods. It is significant to mention that the *L. monocytogenes* species studied were damaged over a prolonged period. The results acquired revealed that the rate of inactivation of the tested compound as a *K*_1_ constant was rapid in *E.* *coli* > *P.* *aeruginosa* > *K.* *pneumoniae* > *S.* *aureus* > *B.* *subtilis* > *L.* *monocytogenes* (Table 4).

### 2.5. Physiological Altering of Bacterial Species

**Figure 3:**
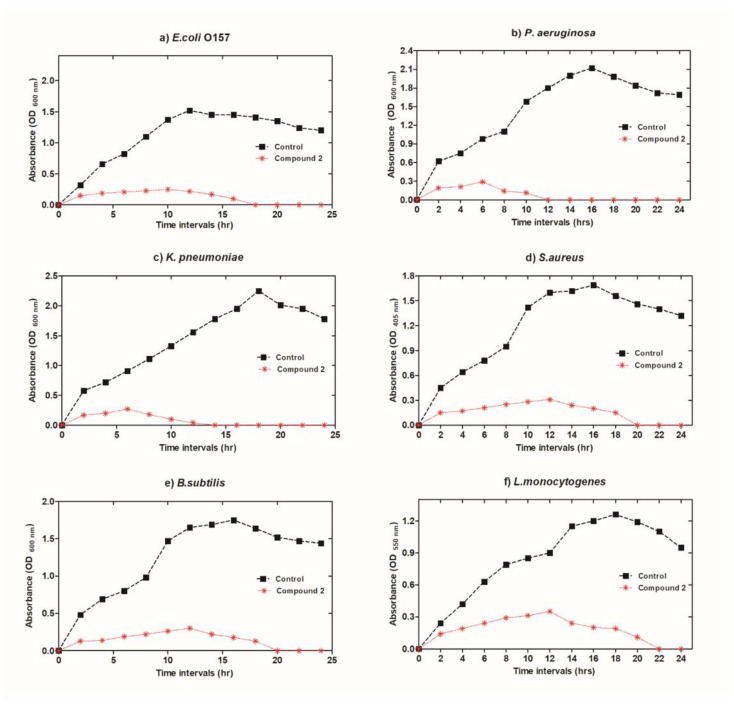
The growth curves of (**a**) *E. coli* O157, (**b**) *P.* *aeruginosa*, (**c**) *K.* *pneumoniae* (**d**) *S.* *aureus*, (**e**) *B. subtilis*, (**f**) *L.* *monocytogenes* before and after subjecting them to the tested compound.

As seen in Figure 3, the results revealed that all the tested bacterial species’ growth rates decreased gradually and significantly after exposure to the new combined effective dose of the compound. The results obtained showed that when comparing the bacterial growth curves in all studied bacteria, the slope of the bacterial growth curve was faster and more significant for *P.* *aeruginosa* bacteria, and conversely, the rate of decrease was lower for *L.* *monocytogenes* species.

**Figure 4:**
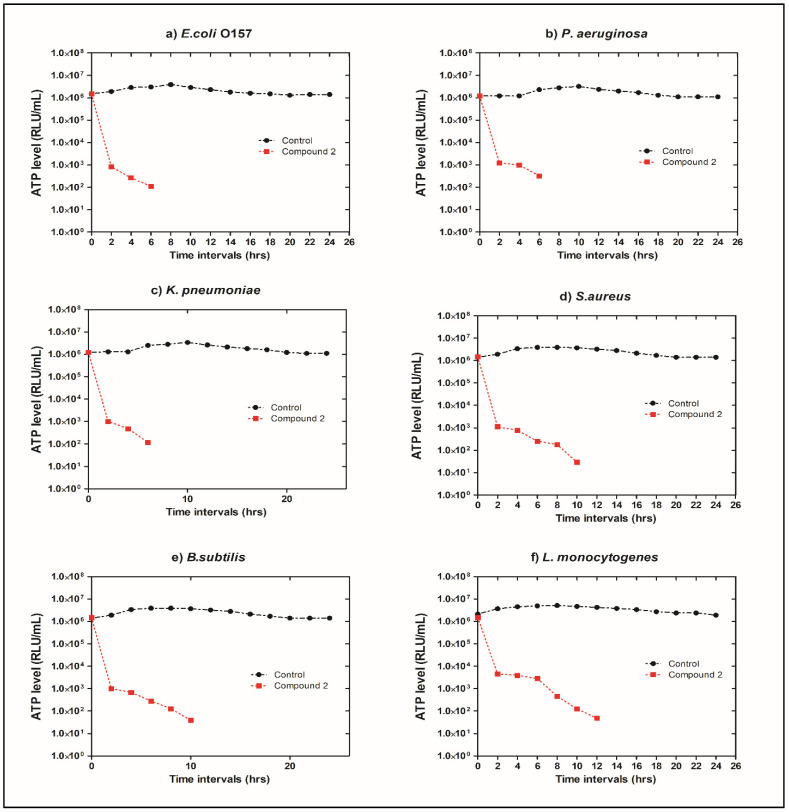
Amounts of ATP produced from (**a**) *E. coli* O157, (**b**) *P. aeruginosa*, (**c**) *K. pneumoniae* (**d**) *S. aureus*, (**e**) *B. subtilis*, (**f**) *L. monocytogenes* before and after subjecting them to the tested compound.

In the same context, the level of ATP is an excellent indicator of the activity and vitality of the bacterial cells and the extent of their ability to grow and cause infection and damage. The results represented in Figure 4 revealed that the level of ATP was significantly decreased in the bacterium *P.* *aeruginosa* compared to that in the other species tested, while the level of decrease was lower in *L.* *monocytogenes*.

### 2.6. Protein Release

**Figure 5:**
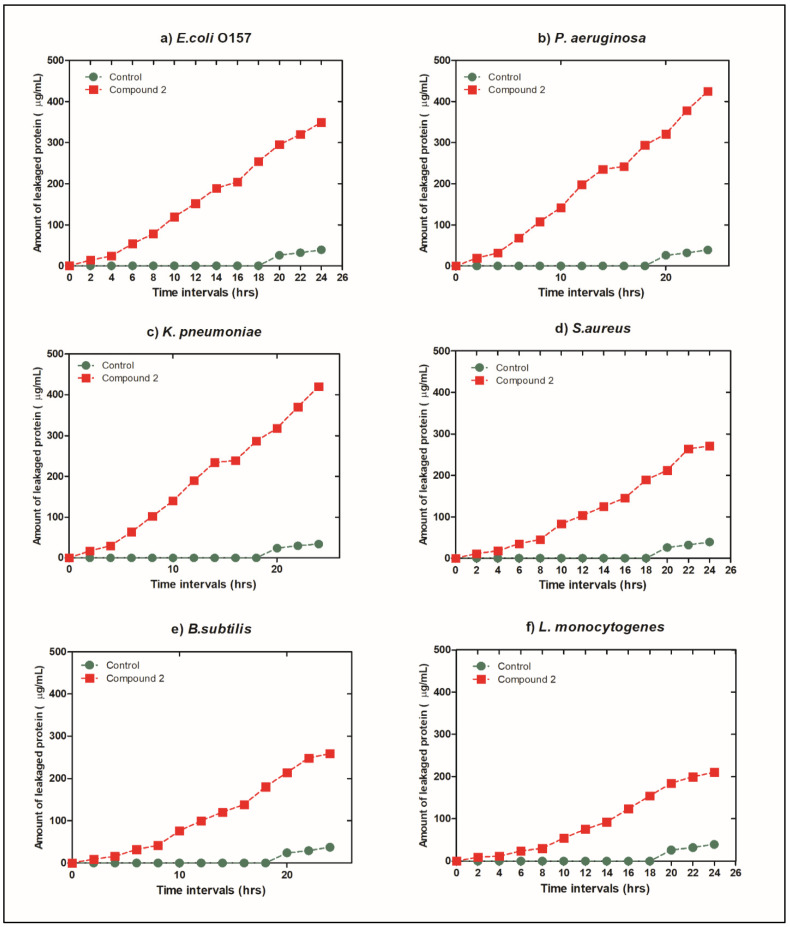
Quantities of protein leakage from (**a**) *E. coli* O157, (**b**) *P. aeruginosa*, (**c**) *K. pneumoniae (***d**) *S. aureus*, (**e**) *B. subtilis*, (**f**) *L. monocytogenes* before and after subjecting them to the tested compound.

In terms of protein leakage from weakened bacterial cells, as seen in Figure 5, the findings showed that after submitting the cells to the successful concentration of the compound, the quantities of protein released increased dramatically. The level of protein released by *P. aeruginosa*, an indicator of Gram-negative bacteria, was increased in which the amounts of liberated protein were greater than for other species (Figure 5). Due to the small cell wall, porous interstitial structures, and the formation of weak lipopolysaccharides, these results corroborate those of Jiang et al. [24], who reported that the protein release rate and quantities from compromised *E. coli* cells were quicker and more meaningful, respectively than those found in *S. aureus*. Therefore, it could be inferred that the studied compound may trigger substantial morphological changes in the microbial cell wall and measurable cell material outflow [25]. Gram-negative bacteria such as *E. coli* and *P. aeruginosa* have less ability to withstand environmental stress, which results in losses or deformation due to a lack of bacterial arrangements. Softly porous bacterial cells are produced by antimicrobial compounds [26].

Conversely, the cell wall of Gram-positive bacterial strains makes up about 90% of the peptidoglycan, which gives their walls more robust mechanical properties. Wher one of these features can enhance the resistance of these microbial species to antimicrobial compounds [27]. Several pieces of evidence indicate that the slope in the bacterial growth curve, as well as the level of released protein as a result of cell destruction, were more significant in Gram-negative bacteria compared to the Gram-positive species, and this is due to the rigid and inflexible structure of the Gram-positive cell wall, which makes it more resistant to bactericidal agents [28].

### 2.7. Toxicological Performance Assay of Compound

Due to the prevalence of antibiotic-resistant species, drug manufacturers are looking for promising alternative biocompatible antibacterial agents that do not show long-term toxicity. Appraising cytotoxic effects is obviously a noteworthy phase in evaluating a potential antimicrobial drug since a helpful drug must be safe and non-toxic for the intended host. By testing its possible toxicity to the bioluminescent marine bacterium ‘*Aliivibriofischeri*,’ the biocompatibility of the studied compound was assessed. In Table 4, experimental approximate EC_50_ % values for the tested compound are listed. The toxicity assay was implemented to verify the newly synthesized compound’s safety, and the result indicated toxicity records at three separate intervals (5, 15, and 30 min), and the results showed that the compound tested had no toxic impact and was safe for humans. The EC_50_ % readings after 5, 15, and 30 min were 247, 235, and 218, respectively. The results also indicated that the tested compound’s EC_50_ % values were higher than 100% at various times, indicating that the compound is non-toxic (Table 5).

Moreover, a MMT assay was applied to measure the compound’s cytotoxic effect by estimating the CC_50_ and safe dose of the tested compound against HEp-2 cell lines; the results showed that the CC_50_ was 0.825 mg/mL, which means that the synthesized compound had no cytotoxic effect on HEp-2 cells.

These results accord well with the results of El Malah et al. [16]. Similarly, cytotoxicity testing has been performed in several other research studies, all of which confirmed that the synthetic triazole did not show long-term toxic effects [29].

### 2.8. Computational Studies

**Figure 6:**
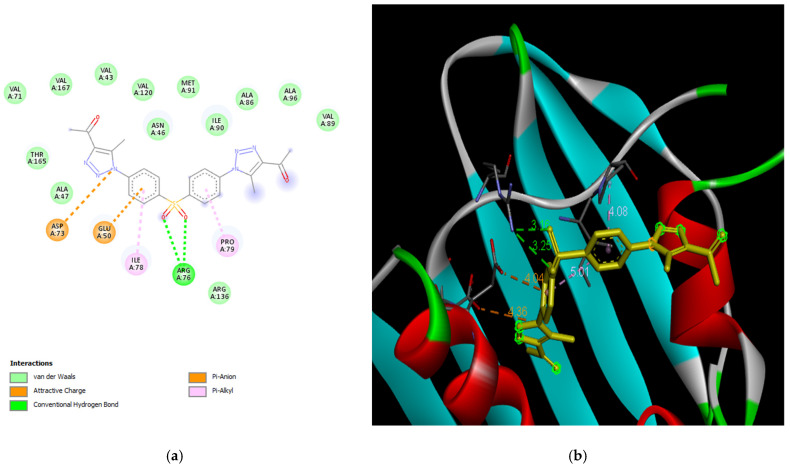
(**a**) 2D representation of the compound docked in the enzyme DNA gyrase pocket (PDB id: 1KZN). (**b**) 3D representation.

Because of their vital role in DNA duplication, topoisomerase enzymes have become a significant focus for drug developers. DNA gyrase is a type II topoisomerase that exists within every bacterium and dictates the DNA topological status. Relaxing the supercoiled DNA is a substantial step for either replication or transcription [30]. Therefore, blocking this step by inhibiting DNA gyrase is the key role of many antibiotics and antimicrobial compounds [31]. In the current study, molecular docking results showed that the compound exhibited good binding affinity, with a high dock score of (−8.8 kcal/mol) recorded against 1KZN protein (DNA gyrase), when compared with the co-crystallized drug clorobiocin (−9.7 kcal/mol), with both sharing some binding positions. Both the docking and experimental results show that the compound stands as a promising antimicrobial drug candidate. The compound showed the number of interactions that stabilize the blockage of the 1 KZN pocket. It could form two hydrogen bonds between the two oxygen atoms of the sulphone group, and could also form the amino acid residue Arg76 with bond lengths of 3.16 and 3.25 Å. An attractive charge interaction between (a) aryl group and Glu50 (b) N in the triazole ring and Asp73 also took place. Another hydrophobic interaction occurred between the two benzene rings and residues pro79 and 78. Those two hydrophobic interactions are also present in the case of clorobiocin presence in the DNA gyrase pocket (Figure 6).

**Figure 7:**
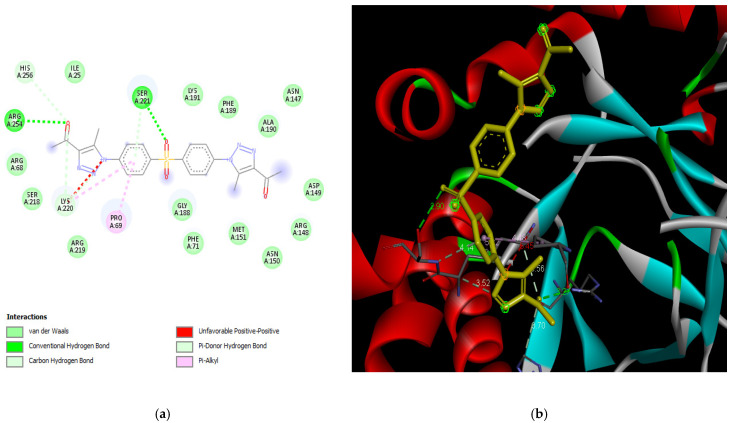
(**a**) 2D representation of the compound docked in the Dihydropteroate synthase (DHPS) pocket (PDB id: 3TYE). (**b**) 3D representation.

Dihydropteroate synthase (DHPS) is a critical enzyme in the biosynthetic machinery of folic acid, which is indispensable for bacterial survival. DHPS catalyzes the reaction of 6-hydroxymethyl-7,8-dihydropterin-pyrophosphate with p-aminobenzoic acid (pABA) to produce the folate intermediate 7,8-dihydropteroate (DHF) [32]. Compounds that compete with pABA and bind to the active site of DHPS inhibit it from preventing bacterial growth. Sulfonamide antibiotics work with a similar mechanism [33]. In the current study, compound **2** was docked against DHPS (PDB ID: 3TYE). Compound **2** could fit in the pocket and make interactions that stabilize its docking in the active site of DHPS. Two hydrogen bonds formed between the O atom of the terminal carbonyl and the residue of Arg254, while the second was among the O of sulphone and Ser221. The bond length was 3.27 and 2.9 Å, respectively. A Pi-interaction type formed between the benzene ring and the pro69 and lys220 residues and the N of triazole and lys220. Another type of H-bond (Pi-donor H-bond) formed between the terminal carbonyl and His256 and Lys220. A possibly unfavorable positive–positive interaction formed between the N of triazole and lys220. Like the co-crystallized inhibitor (sulphoneamide compound) complexed to3TYE, the compound shared various interactions with the residues of ser221, lys220, pro69, and arg254 in strength and nature. The ability of the compound to dock and stabilize in two pockets of two different vital microbial enzymes makes it a promising lead for antimicrobial drugs (Figure 7).

## 3. Materials and Methods

### 3.1. Chemistry

#### 3.1.1. Experimental Instrumentation

All melting points were determined on an electrothermal apparatus and are uncorrected. IR spectra were recorded (KBr discs) on a Shimadzu FT-IR 8201 PC spectrophotometer. ^1^H NMR and ^13^C NMR spectra were recorded in (CD_3_)_2_SO solutions on a BRUKER500 FT-NMR system spectrometer, and chemical shifts are expressed in ppm units using TMS as an internal reference. Mass spectra were recorded on a GC-MS QP1000 EX Shimadzu. Elemental analyses were carried out at the Microanalytical Center of Cairo University.

#### 3.1.2. 1,1′-((Sulfonylbis(4,1-phenylene))bis(5-methyl-1H-1,2,3-triazole-1,4-diyl))bis(ethan-1-one) (**2**)

A mixture of 4,4′-sulfonylbis(azidobenzene) (3 g, 10 mmol) and Acetyl acetone (2 mL, 20 mmol) were stirred under reflux in ethanol containing sodium methoxide (0.5 g, 10 mmol) for 5 h; the resulting solid that formed after cooling was collected and recrystallized from ethanol to give white crystals, m.p. 248–250 °C; yield (95%); FT-IR (KBr, cm^−1^): *v* 2919, 2852 (CH), 1617 (C=C); ^1^H NMR (500 MHz, DMSO-d*6*): *δ* 2.36 (s, 6H, 2CH_3_), 2.42 (s, 6H, 2CH_3_), 7.75 (d, 4H, *J* = 10.0 Hz, ArH), 8.11 (d, 4H, *J* = 10.0 Hz, ArH);^13^C-NMR (100 MHz, DMSO-d*6*): *δ* 9.74, 27.69, 126.65, 129.33, 138.10, 139.18, 141.41, 142.98, 193.33; MS: *m*/*z* [%]: 464 (M^+^, 17), 450 (22), 448 (18), 378 (3), 273 (21), 177 (100), 165 (18), 77 (90), 55 (45). Analysis: calcd. For C_22_H_20_N_6_O_4_S (464): C, 56.89; H, 4.43; N, 18.09% found: C, 56.92; H, 4.39; N, 18.02%.

### 3.2. Biology

#### 3.2.1. Evaluation of Antimicrobial Properties of the Synthesized Compound

##### Antibacterial Susceptibility Testing (AST)

The antimicrobial efficacy and zone of inhibition (ZOI) diameters of the synthesized compound were assessed using the Kirby–Bauer agar diffusion assay (disc and well diffusion) against six different kinds of pathogenic bacteria, including three Gram-negative (*E. coli* O157:H7 ATCC 35150, *Pseudomonas aeruginosa* ATCC 10145, and *Klebsiella pneumonia* ATCC 13889) and three Gram-positive (*Staphylococcus aureus* ATCC 43300, *Bacillus subtilis* ATCC 4342*,* and *Listeria monocytogenes* ATCC 25152) bacteria. On plates of nutrient agar for bacterial strains, each strain’s pure colonies were sub-cultured and incubated at 37 °C for 24 h. McFarland standard 0.5 microbial suspensions (1.5 × 10^6^ CFU/mL) in the saline tube were prepared using the McFarland reader [34].

The above-mentioned checked strains were carefully dispensed with standardized inoculum on the Mueller Hinton Agar (MHA) plates’ surface. Under aseptic circumstances, the disc-diffusion assay was conducted. The sterile discs were saturated with 50 μL of the newly synthesized compound at a 20 mg/mL concentration and kept in aseptic surroundings for drying. The saturated discs were then placed tightly on the surface of the MHA plates using sterile forceps. In the well-diffusion assay, the wells (6 mm in diameter) in the MHA agar medium with a deep layer were punched using a sterile cork borer. A particular volume of 50 µL of the newly synthesized compound was aseptically dispensed into each well. The negative regulation was sterile purified water, and antibiotic resistance (vancomycin, Ciprofloxacin) discs were applied as positive controls. After this, all plates were inverted and placed in an incubator at 37 °C overnight. The zone of inhibition (ZOI) values across the discs and wells were recorded using a Vernier caliper [35]. In addition, the IC_50_ values and log IC_50_ record (concentrations that can eliminate fifty percent of cell viability) for the specific compound were calculated in GraphPad Prism using nonlinear dose-response modeling., following Lavorgna et al. [36].

### 3.3. Determination of Minimum Inhibitory Concentrations

The minimum inhibitory concentrations (MICs) of the synthesized compound were assessed using a macrodilution assay. The synthesized compound concentration range obtained was from 10–50 mg/mL. In three tubes, the positive control (media containing antibiotic inoculum), negative control (media containing inoculum), and synthesized compound solution (media containing the doses of the synthesized compound) were added. After several retention times, ranging from 10–30 min, one mL of each tube was transferred to sterile plates, and the appropriate volume of melted nutrient agar medium was poured. Then, all inoculated plates were incubated at 37 °C for 24 h. By comparing the positive and negative control tubes, MIC values were determined. MICs were characterized as the lowest concentration with no growth in plates. The findings were presented as the mean values for two different replicates. CLSI identified the breakpoints of MICs (μg/mL) for antibiotics [17].

### 3.4. Pseudo-First-Order Kinetic Modeling

To estimate the destructive values (*k*1) of each bacterial species considered, kinetic modeling was utilized to identify the number of viable cells bearing the required concentration of the synthesized compound tested over different retention times (Nt) to the initial cell numbers (N0), by using the equation below (1) [37].

(1) $$\log\left(q_{e}-q_{t}\right)=\log q_{e}-\frac{K_{1}t}{2.303}$$

where *k*_1_ (1 min^−1^) is the rate of inactivation; *q_e_* (mg·g^−1^), and *q_t_* (mg·g^−1^) are the amounts adsorbed at equilibrium and at time *t* (min), respectively. A straight line of ln (*q_e_ − q_t_*) versus *t* suggested that this kinetic model was applied to the data [38].

### 3.5. Physiological Altering of Bacterial Strains

Functional variability in the growth and structure of the examined bacteria were investigated by injecting 100 μL of bacterial cells into two tubes containing 50 mL of sterile Typricase soy broth. The tested compound’s effective concentration (50 mg/mL) was inserted into one of these tubes, while another volume (without any compound) was tested as a negative control. All tubes were placed in a shaking incubator at 37 °C, shaking at 200 rpm, and samples were collected over 24 h (n = 12 readings) every 2 hrs [19].

### 3.6. Bacterial Growth Rate

One ml was obtained from each specified tube to estimate optical density at 600 nm using the spectrophotometer to calculate each studied bacterial strain [39].

### 3.7. Estimation of Amounts of Released Protein

The quantities of protein extracted from destroyed cells were assessed with the Coomassie blue assay [40,41].

### 3.8. ATP Bioluminescence Assay

When ATP levels decrease rapidly after cell death, ATP represents a considerable variation in cell viability. Through quantifying the extracellular ATP levels using the luciferin-luciferase process, bacterial activities and viabilities were measured. A 30 μL ATP aliquot solution was combined with a 270 μL luciferin-luciferase aliquot mixture throughout this simple assay, and the mixture was ultimately applied to the optical detection cells. Using the ATP luminometer, the luminescence patterns and luminescence intensity were estimated and distinguished as relative light units (RLU) [42].

### 3.9. In Vitro Toxicological Performance Assay of the Tested Compounds

Using a Microtox^®^ Model 500 (M500) analyzer (Modern Water, New Castle, DE, USA), the toxic effects of the compound were verified to ensure their safe and productive use for medicinal and biomedical applications without any harmful effects on individuals. The toxicity level was measured at the maximum concentration (50 mg/mL) of each compound tested [43].

The cytotoxicity assay on the HEp-2 cell lines by a colorimetric MTT assay was applied to determine the percentage of surviving cells. MTT (3,4,5-(dimethylthiazol-2-yl) 2-5-diphenyl tetrazolium bromide) is reduced to its violet formazan product by metabolically viable cells. The MTT assay was utilized to determine the compound’s cytotoxicity. As indicated above, the cells were subjected to multiple compound concentrations and kept in the incubator for 24 h at 37 °C. The MTT procedure was conducted when the culture medium was substituted with a new medium. At 570 nm, the purple color product was measured. [1−Absorbance of treated cells/Absorbance of untreated cells] × 100 = percentage cell viability. The remaining absorbance from the wells containing untreated cells (negative control) was applied to determine the vitality of the cells. Camptothecin (5 mM, 20 L) was a positive control [44] (Soysa et al., 2014).

### 3.10. Computational Studies

DNA gyrase and Dihydropteroate Synthase X-ray protein structures were downloaded from the protein data bank (www.rcsb.org, accessed on 30 June 2021) PDB ID: 1KZN, 3TYE respectively. For both proteins, water molecules and co-crystalized ligands were removed, then the prepared structures were saved as PDB files by BioviaDiscoverstudio2021 for further docking steps. The CB-DOCK webserver was used for cavity detection, and molecular docking [45] was accessed via (http://clab.labshare.cn/cb-dock/php/, accessed on 30 June 2021) to perform a docking with its default standard protocol. The compound was docked against 1KZN at Cavities volume 379, grid box dimensions x: 20.15, y: 23.67, z: 35.5 and box size x: 27, y: 27, z: 27. The compound was docked against 3TYE using the same server at Cavities volume 739, grid box dimensions x: −79.12, y: 90.52, z: 96.84, and box size x: 27, y: 27, z: 27.

## 4. Conclusions

1,1′-((sulfonylbis(4,1-phenylene))bis(5-methyl-1H-1,2,3-triazole-1,4-diyl))bis(ethan-1-one) (**2**) was synthesized via the reaction of 4,4′-sulfonylbis(azidobenzene) (**1**) with acetylacetone and sodium methoxide in ethanol. Its chemical structure was inferred from correct microanalytical and chemical data. It was also screened to assess its antibacterial efficacy. The reported results revealed that the tested compound exhibited outstanding antimicrobial properties against Gram-negative and positive strains. Hence, it might be applied in biomedical and pharmacological uses.

## Figures and Tables

**Table 1:** Inhibition zone testing and ZOI diameters of the synthesized compound against tested harmful bacterial strains.

Tested Bacterial Pathogens	Width of ZOI (mm)					
	Synthesized Compound		Vancomycin		Ciprofloxacin	
	Disc Diffusion	Well Diffusion	Disc Diffusion	Well Diffusion	Disc Diffusion	Well Diffusion
*E. coli* O157	22 ± 0.14	24 ± 0.14	15 ± 0.22	17 ± 0.24	16 ± 0.23	18 ± 0.14
*P. aeruginosa*	23 ± 0.28	26 ± 0.24	17 ± 0.17	19 ± 0.11	18 ± 0.09	20 ± 0.05
*K. pneumoniae*	25 ± 0.23	27± 0.12	16 ± 0.15	18 ± 0.25	17 ± 0.25	19 ± 0.22
*S. aureus*	19 ± 0.26	22 ± 0.21	13 ± 0.24	15 ± 0.15	12 ± 0.21	14 ± 0.14
*B. subtilis*	17 ± 0.16	20 ± 0.23	12 ± 0.18	14 ± 0.16	10 ± 0.14	12 ± 0.13
*L. monocytogenes*	18 ± 0.22	21 ± 0.16	10 ± 0.20	13 ± 0.18	8 ± 0.12	11 ± 0.08

**Table 2:** Estimated MIC values of reference antibiotics drugs used.

Tested Bacterial Pathogens	Reference Antibiotics Drugs Used	
	Vancomycin	Ciprofloxacin
*E. coli* O157	50 µg/mL	20 µg/mL
*P. aeruginosa*	50 µg/mL	20 µg/mL
*K. pneumonia*	40 µg/mL	30 µg/mL
*S. aureus*	20 µg/mL	50 µg/mL
*B. subtilis*	30 µg/mL	40 µg/mL
*L. monocytogenes*	30 µg/mL	40 µg/mL

**Table 3:** The calculated IC_50_, Log IC_50,_ and R^2^ of the studied compound.

Tested Bacterial Pathogens	Compound		
	IC_50_ (µM)	Log IC_50_ (µM)	R^2^
*E. coli* O157	22.42	1.35	0964
*P. aeruginosa*	11.11	1.02	0.945
*K. pneumonia*	16.54	1.21	0.936
*S. aureus*	25.31	1.40	0.978
*B. subtilis*	26.66	1.43	0.983
*L. monocytogenes*	31.38	1.50	0.965

**Table 4:** Kinetic values (*K*_1_ (min^−1^)) of pseudo-first-order calculation for the newly synthesized compound’s inactivation of tested bacterial strains.

Tested Bacterial Pathogens	The Synthesized Compound (50 mg/mL)	
	*K*_1_	*R* ^2^
*E. coli* O157	0.4273	0.9688
*P. aeruginosa*	0.3702	0.9737
*K. pneumonia*	0.3001	0.9931
*S. aureus*	0.2981	0.9812
*B. subtilis*	0.2478	0.9888
*L. monocytogenes*	0.1847	0.9886

**Table 5:** The values of Microtox^®®^ EC_50_ percent concentrations of the studied compound.

Studied Compound	EC_50_ % Conc.	EC_50_% Degree	Toxicity Level
**Compound after 5 min**	247	0–19	Extremely toxic
		20–39	Very toxic
**Compound after 15 min**	235	40–59	Toxic
		60–79	Moderately toxic
**Compound after 30 min**	218	≥100	Non-toxic and safe

## Data Availability

Not applicable.

## Supplementary Materials

The following are available online, Chart 1: 1HNMR spectrum of compound 2, Chart 2: C13NMR spectrum of compound 2.

## Sample Availability

Samples are available from the corresponding author.
