# Supplementary material for: Antibacterial Activities and Molecular Docking of Novel Sulfone Biscompound Containing Bioactive 1,2,3-Triazole Moiety
Source: Molecules. 2021 Aug 9;26(16):4817. doi: 10.3390/molecules26164817 (PMC8399954; doi:10.3390/molecules26164817)
Supplement: Supplementary file 1 [file molecules-26-04817-s001.zip › molecules-1304128-supplementary.pdf]

Antibacterial Activities and Molecular Docking of Novel Sulfone Biscompound Containing Bioactive 1,2,3-Triazole Moiety

Huda R. M. Rashdan [1,*], Ihsan A. Shehadi [2], Mohamad T. Abdelrahman [3] and Bahaa A. Hemdan [4]

[1] Chemistry of Natural and Microbial Products Department, Pharmaceutical and Drug Industries Research Division, National Research Centre, Dokki, Cairo 12622, Egypt

[2] Chemistry Department, College of Science, University of Sharjah, Sharjah 27272, United Arab Emirates; ishehadi@sharjah.ac.ae

[3] Radioisotopes Department, Nuclear Research Centre, Egyptian Atomic Energy Authority, Cairo 12311 Egypt; mohamadt.abdelrahman@gmail.com

[4] Water Pollution Research Department, Environmental Research Division, National Research Centre, 33 El Buhouth Street, Cairo 12622, Egypt; be.hemdan@iitg.ac.in

* Correspondence: hr.rashdan@nrc.sci.eg

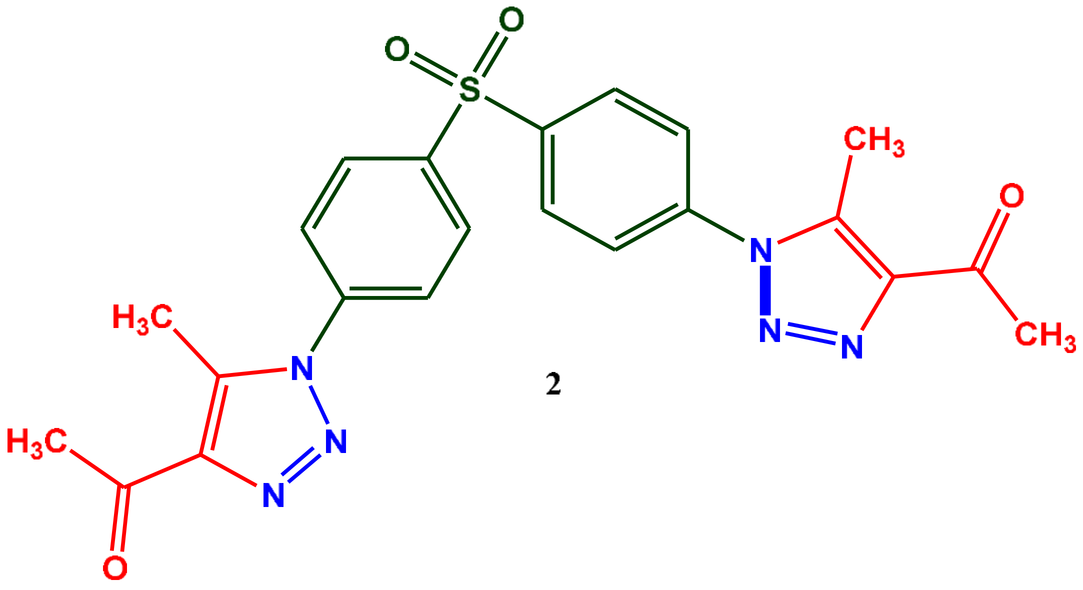

2

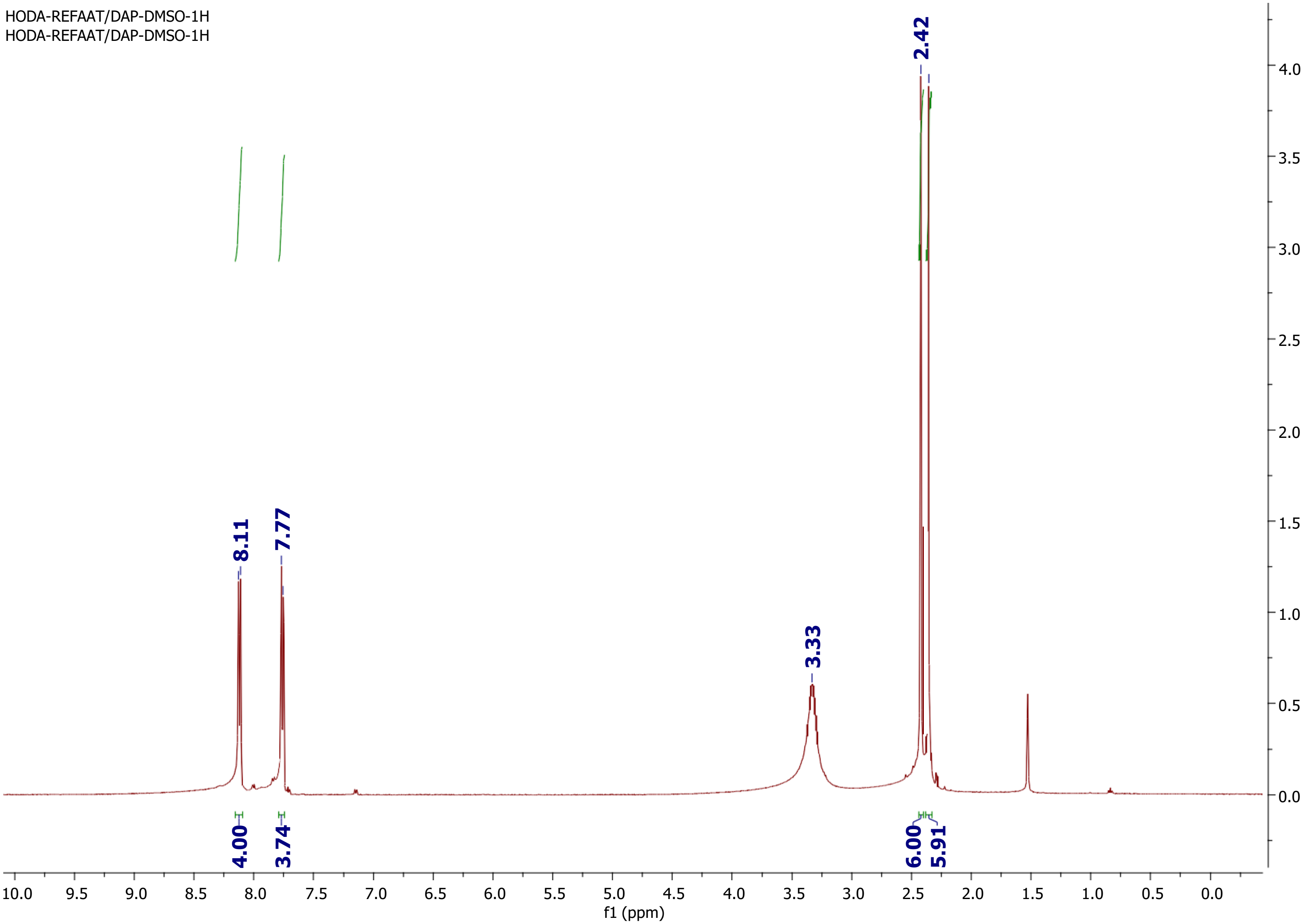

HODA-REFAAT/DAP-DMSO-1H
HODA-REFAAT/DAP-DMSO-1H
8.11
7.77
3.33
2.42
4.00
3.74
6.00
5.91
f1 (ppm)

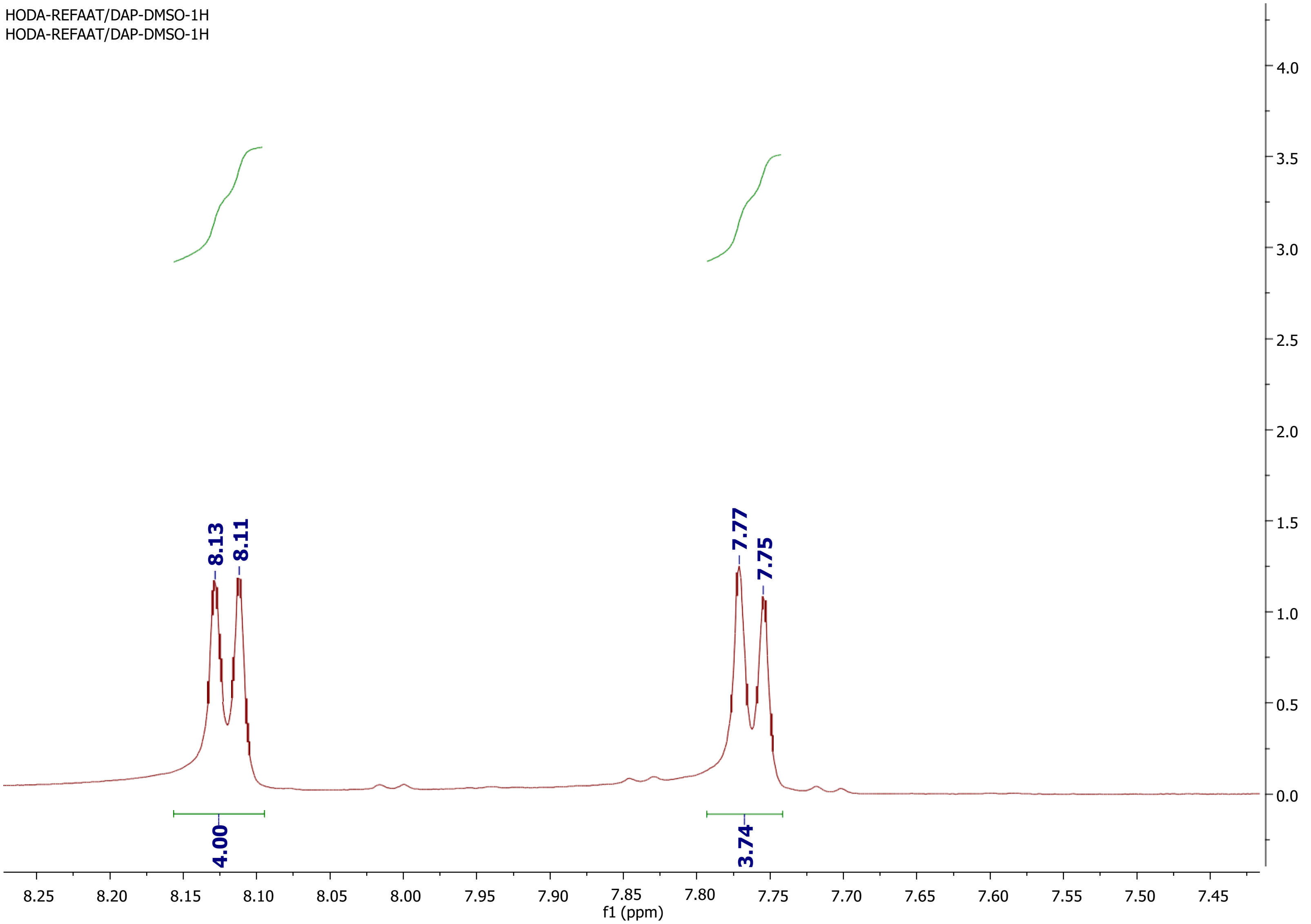
HODA-REFAAT/DAP-DMSO-1H
HODA-REFAAT/DAP-DMSO-1H
8.13
8.11
7.77
7.75
4.00
3.74
f1 (ppm)

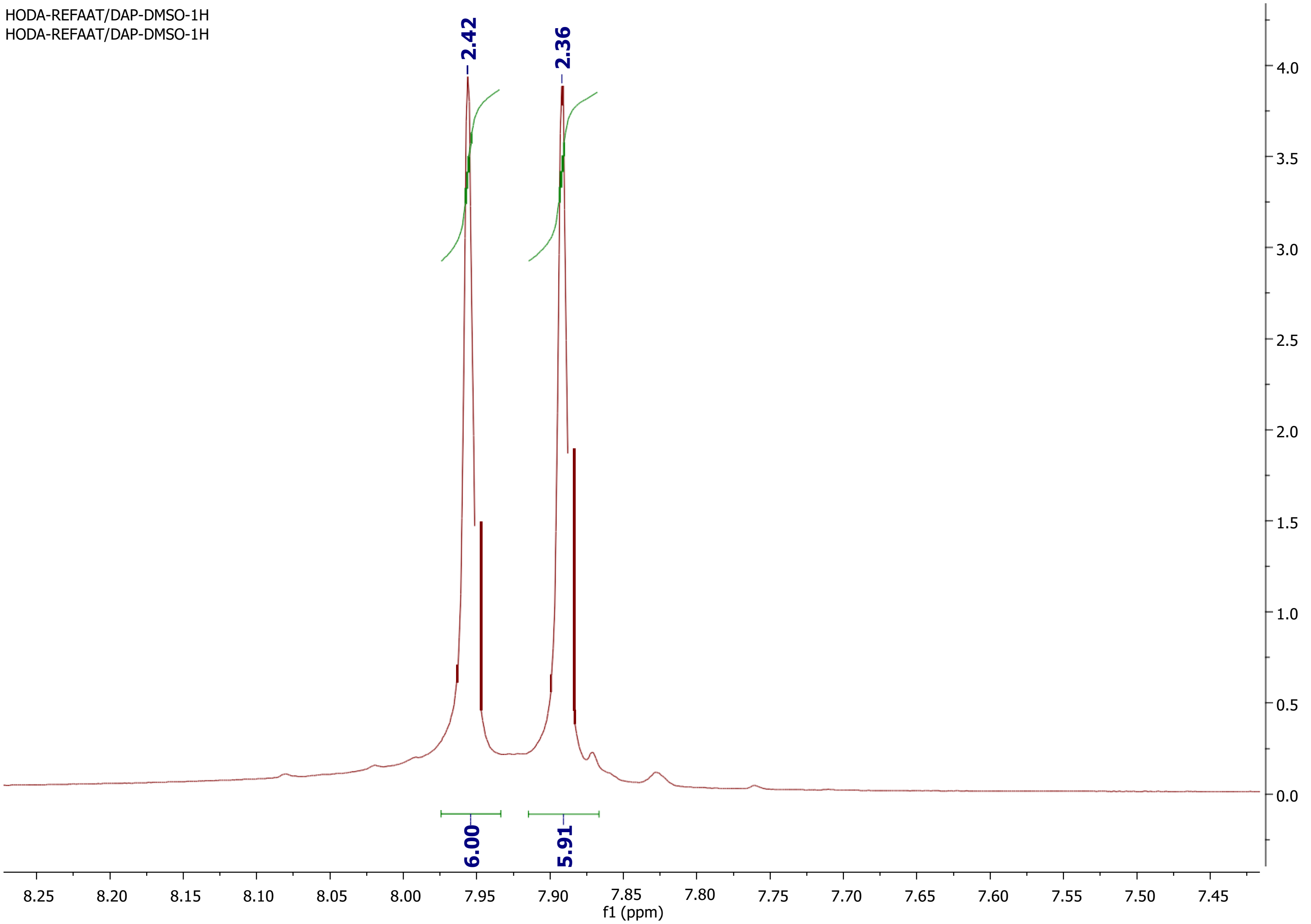

HODA-REFAAT/DAP-DMSO-1H
HODA-REFAAT/DAP-DMSO-1H
2.42
2.36
6.00
5.91
f1 (ppm)

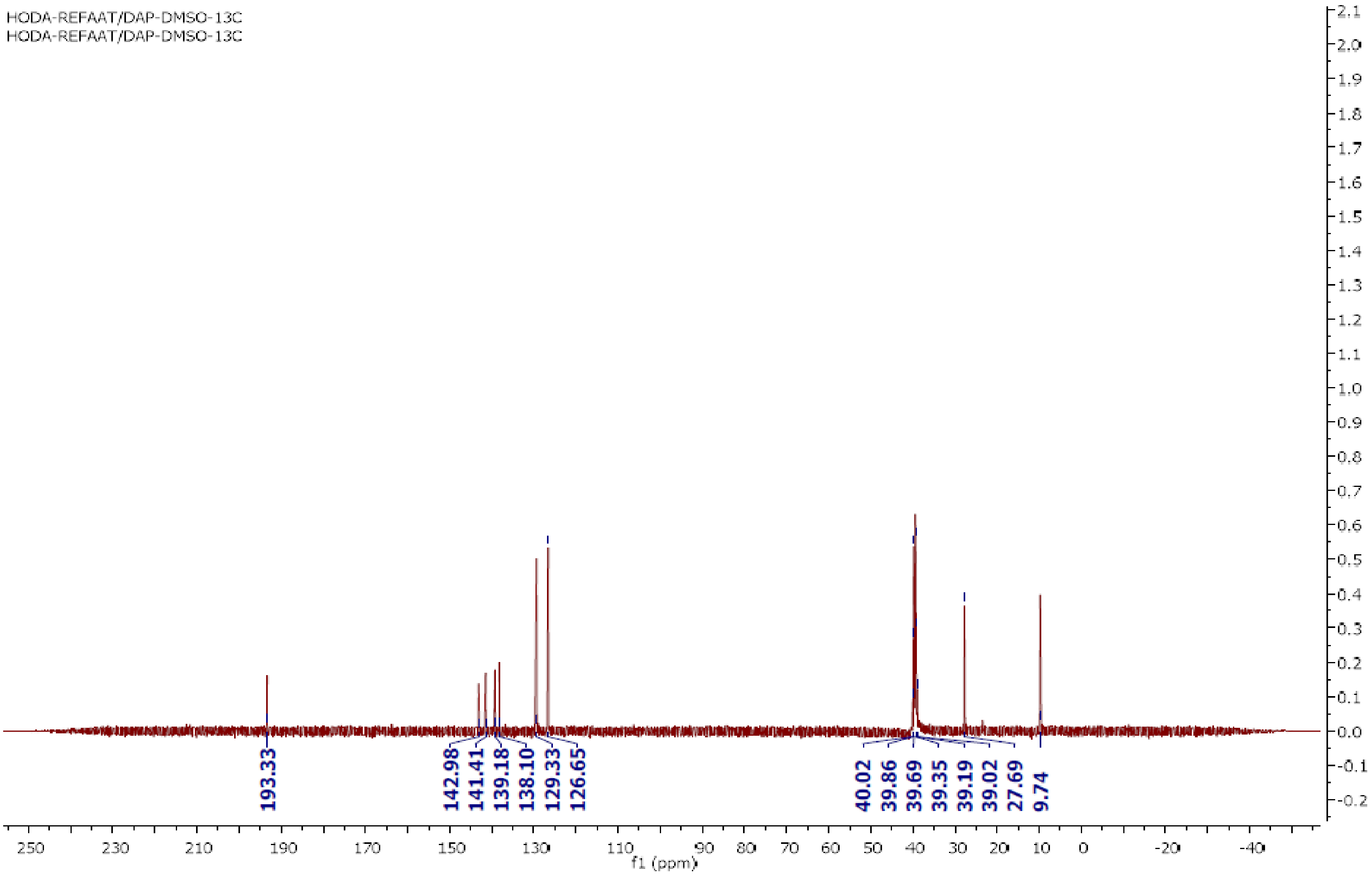
HODA-REFAAT/DAP-DMSO-13C
HODA-REFAAT/DAP-DMSO-13C
193.33
142.98
141.41
139.18
138.10
129.33
126.65
40.02
39.86
39.69
39.35
39.19
39.02
27.69
9.74
f1 (ppm)
